# Beyond Sleeping Disorders, the Role of Melatonin in Skin Diseases and Emerging Applications in Dermatology and Topical Therapy

**DOI:** 10.3390/gels11110860

**Published:** 2025-10-27

**Authors:** Jesus A. Simon, Celia Serrano, Dinesh Kumar, Brayan J. Anaya, Liliana Bautista, Carlos Torrado-Salmerón, Dolores R. Serrano

**Affiliations:** 1 Pharmaceutics and Food Technology Department, School of Pharmacy, Universidad Complutense de Madrid, Plaza Ramón y Cajal s/n, 28040 Madrid, Spain; 2 Department of Pharmaceutical Engineering and Technology, Indian Institute of Technology (BHU), Varanasi 221005, India; 3 Institute of Industrial Pharmacy, School of Pharmacy, Complutense University of Madrid, 28040 Madrid, Spain

**Keywords:** melatonin, skin, cancer, topical formulations, gels, creams, SNEDDS patch, antioxidant, anti-inflammatory, antiaging, wound healing, UV radiation

## Abstract

Melatonin, traditionally recognized for its role in regulating circadian rhythms and sleep, has emerged as a multifunctional molecule with significant implications in dermatology. Melatonin is described here as a pleiotropic, context-dependent modulator with antioxidant-related and immunomodulatory actions that are supported by both direct chemical scavenging in cell-free systems and indirect, enzyme-mediated effects in cells and tissues; its antitumor activity in dermatology is primarily preclinical and remains to be confirmed in large clinical trials. Melatonin protects skin cells from oxidative stress, UV radiation, and environmental damage by directly scavenging free radicals and activating endogenous defense systems. It also modulates immune responses, making it relevant in inflammatory dermatoses such as atopic dermatitis, while promoting tissue repair in wound healing and reducing signs of skin aging. Particular attention is given to topical formulations, including gels, creams, and patches, which enable localized delivery, improved skin penetration, and reduced systemic side effects. The review also discusses ongoing clinical trials, delivery technologies, and the potential for combinatorial therapies with established dermatological agents.

## 1. Introduction

The skin, our body’s largest organ, serves as a dynamic interface between the internal milieu and the external environment. Its health and integrity are paramount for overall well-being, yet it is constantly challenged by a myriad of factors, including environmental stressors, intrinsic aging, and various dermatological conditions. In the quest for effective dermatological interventions, melatonin has emerged as a promising molecule with a diverse range of biological activities.

Melatonin, a hormone-derived metabolite with multifunctional biological roles primarily synthesized by the pineal gland, is well-known for its role in regulating circadian rhythms and sleep. However, its presence and synthesis in various peripheral tissues, including the skin, have underscored its multifunctional nature. In the realm of dermatology, melatonin’s antioxidant, anti-inflammatory, and immunomodulatory properties have garnered significant attention, positioning it as a potential therapeutic agent for a spectrum of skin disorders [1].

This literature review aims to provide a comprehensive overview of the dermatological applications of melatonin. We will delve into the intricate mechanisms through which melatonin exerts its effects on the skin, elucidating its role in mitigating oxidative stress, modulating inflammatory responses, and influencing skin physiology. The scope of this review encompasses a wide array of dermatological applications, ranging from the prevention and treatment of skin aging to the management of inflammatory dermatoses such as acne, atopic dermatitis, and psoriasis [2]. We will also explore the role of melatonin in wound healing, skin cancer protection, and other relevant dermatological conditions. Furthermore, this review will shed light on the various formulations of melatonin available for dermatological use, including topical applications with a main focus on gels and oral supplements, and discuss their efficacy and safety profiles [3,4].

### 1.1. Melatonin’s Mechanisms of Action in the Skin

Melatonin, chemically known as N-acetyl-5-methoxytryptamine, is an indoleamine derived from the essential amino acid tryptophan. Its key structural features include an indole ring, a methoxy group (-OCH_3_) at the 5th carbon position, and an N-acetyl side chain (-NHCOCH_3_). This lipophilic structure allows melatonin to readily cross cell membranes, including the blood–brain barrier. Melatonin primarily exerts its effects by binding to specific melatonin receptors, namely MT1 and MT2, which are G protein-coupled receptors (GPCRs) [5,6]. These receptors are widely distributed throughout the body, including the brain’s suprachiasmatic nucleus (SCN), the heart, blood vessels, the immune system, and the gastrointestinal tract. Activation of these receptors triggers intracellular signaling cascades, leading to various physiological effects, most notably the regulation of the sleep–wake cycle (circadian rhythm). In the SCN, MT1 and MT2 receptor activation plays a crucial role in synchronizing the body’s internal clock with the external light–dark cycle [7].

Beyond its receptor-mediated effects, melatonin is also a potent antioxidant [8,9]. It can directly scavenge a wide range of free radicals and reactive oxygen and nitrogen species, protecting cells throughout the body from oxidative damage. Furthermore, it stimulates the production of endogenous antioxidant enzymes like superoxide dismutase (SOD) and glutathione peroxidase (GPx). In addition to these primary mechanisms, melatonin can also modulate the activity of certain enzymes, interact with binding proteins such as G protein-coupled membrane receptors (MT1, MT2, MT3) and intracellular proteins (Calmodulin, Protein Kinase C, Nuclear proteins, Glycolytic proteins), and regulate gene expression, contributing to its diverse physiological roles [10]. Collagen degradation is a major contributor to the visible signs of skin aging, including dryness, roughness, and wrinkle formation. This decline in collagen content is closely associated with reduced mitochondrial synthesis of proline, a key amino acid essential for collagen production. Although the precise mechanisms underlying melatonin’s anti-aging effects remain incompletely understood, recent evidence suggests that melatonin supplementation can mitigate age-related impairments in mitochondrial function. In aging skin, levels of mitophagy, NADK2 (a key enzyme in mitochondrial proline synthesis), proline, and collagen are markedly reduced, whereas oxidative stress is elevated. Melatonin administration has been shown to upregulate mitophagy, enhance NADK2 expression, and restore proline synthesis, thereby promoting collagen regeneration and alleviating the structural and functional manifestations of cutaneous aging [11].

### 1.2. Antioxidant Properties and Their Impact on Cutaneous Oxidative Stress

In line with current redox biology, direct radical scavenging by low-molecular-weight compounds is not considered a significant antioxidant mechanism in vivo; most intracellular protection is mediated by enzymatic systems (e.g., SOD, CAT, GPx) and their regulation. Thus, melatonin’s antioxidant effects in skin are best viewed as indirect, chiefly through upregulation and preservation of endogenous antioxidant defenses (including Nrf2-linked programs), while its robust “scavenger” activity is documented in acellular systems but remains unproven and theoretically unlikely in living tissues [12,13].

The antioxidant efficacy of melatonin is context-dependent, manifesting across a spectrum of biological systems such as the following:

1. Placental Antioxidant Defense: During gestation, the placenta, a critical interface for fetal development, synthesizes melatonin to counteract oxidative stress. This placental melatonin production protects the developing fetus from ROS/RNS-mediated damage, ensuring optimal fetal growth [14].
2. Cutaneous Photoprotection: In the skin, melatonin undergoes enzymatic and non-enzymatic metabolism to several biologically active indolic and kynuric derivatives, including N¹-acetyl-N²-formyl-5-methoxykynuramine (AFMK) and N¹-acetyl-5-methoxykynuramine (AMK). Contrary to earlier assumptions that AFMK was an inert end product, it is now recognized as a reactive intermediate that can participate in secondary redox reactions and decompose to yield formic acid, a metabolite with potential cytotoxic implications when locally accumulated. These kynuramine derivatives are part of the so-called melatoninergic antioxidant cascade, which contributes to both the antioxidant and signaling roles of melatonin but also introduces possible pro-oxidant chemistry under certain conditions [13,15]. AFMK retains and amplifies the antioxidant potential of melatonin, participating in the so-called “antioxidant cascade” by neutralizing highly reactive free radicals, particularly the hydroxyl radical, with proven efficacy in in vitro, in vivo, and computational models. Multiple studies have demonstrated that AFMK protects biological macromolecules (DNA, proteins, and lipids) from radiation-induced oxidative damage, attenuates inflammatory processes, and reduces oxidative stress in conditions such as acute pancreatitis, while also modulating apoptotic pathways that favor the orderly elimination of damaged cells. Furthermore, clinical investigations have reported altered AFMK levels in breast cancer patients, linking them to tumor progression parameters and circadian risk factors, suggesting potential use as a biomarker and possible therapeutic target. Collectively, AFMK emerges as a key component of the cellular defense network initiated by melatonin, with both protective and regulatory implications in oxidative stress, inflammation, and cancer [16,17].
3. Mitochondrial ROS Scavenging: Mitochondria, the cellular bioenergetic centers, are primary targets of oxidative damage. Melatonin mitigates mitochondrial ROS generation and preserves mitochondrial function, thereby preventing mitochondrial dysfunction and apoptosis [18]. However, evidence that melatonin directly scavenges radicals is unequivocal in acellular systems, but translation to in vivo conditions is debated; many reported benefits likely involve upregulation of endogenous antioxidant defenses and mitochondrial effects rather than bulk radical quenching. Likewise, melatonin behaves as an immune buffer, not a universal “booster,” showing stimulatory effects under immunosuppression and anti-inflammatory effects in hyperinflammatory states [13].
4. Neuroprotective Antioxidant Mechanisms: Within the central nervous system, melatonin’s lipophilic properties facilitate its access across the blood–brain barrier. It attenuates neuronal oxidative stress, thereby conferring neuroprotection against neurodegenerative diseases [14,19].

#### 1.2.1. Anti-Inflammatory Effects and Modulation of the Skin’s Immune System

Melatonin modulates the production of pro-inflammatory cytokines, including tumor necrosis factor-alpha (TNF-α), interleukin-1 beta (IL-1β), and interleukin-6 (IL-6), leading to a reduction in systemic and local inflammatory responses. Concurrently, melatonin enhances the synthesis of anti-inflammatory mediators, such as interleukin-10 (IL-10), thereby promoting resolution of inflammation and tissue homeostasis [20].

Furthermore, melatonin influences the activity of various immune cell populations, including macrophages and lymphocytes, modulating their responsiveness to inflammatory stimuli. This immunomodulatory role contributes to the fine-tuning of inflammatory cascades and prevents excessive immune activation [14].

#### 1.2.2. Interaction with Melatonin Skin Cell Receptors

The skin, an organ with an autonomous melatonin system, expresses both melatonin synthesis machinery and melatonin receptors, primarily MT1 and MT2. These receptors are distributed across various cutaneous cell types, including keratinocytes, fibroblasts, melanocytes, and hair follicle stem cells, thereby enabling melatonin to exert pleiotropic effects within the skin [10,21]. Activation of MT1 receptors is associated with robust antioxidant defense, modulation of inflammatory responses, and protection against ultraviolet radiation-induced damage. Furthermore, MT1 receptors play a role in hair follicle regulation, stimulating hair follicle stem cell proliferation by prolonging the anagen phase of the hair growth cycle.

While expressed to a lesser extent than MT1 receptors, MT2 receptors contribute to the antioxidant and anti-inflammatory effects of melatonin and also participate in the regulation of skin pigmentation [22].

- The interaction between melatonin and its dermal receptors holds significant clinical implications for the development of therapeutic strategies targeting various skin conditions, such as skin aging, as melatonin may mitigate premature skin aging by protecting against oxidative stress and inflammation [14].
- Atopic Dermatitis, where melatonin can alleviate inflammation and pruritus [23].
- Alopecia due to stimulation of hair growth [24].
- Protective functions of melatonin in skin cancer [3].

Melatonin exerts its effects through binding to specific membrane receptors, MT1 and MT2, as well as nuclear receptors, RORα and RORγ. These receptors are expressed in various skin cells, including keratinocytes, melanocytes, and immune cells. Activation of melatonin receptors triggers intracellular signaling pathways, including the cAMP/PKA pathway, the MAPK pathway, and the PI3K/Akt pathway, which mediate the diverse biological effects of melatonin. Understanding the role of melatonin receptors and signaling pathways in skin cancer is crucial for developing targeted therapies [3].

## 2. Dermatological Uses of Melatonin

### 2.1. Skin Aging

Melatonin also modulates the cutaneous inflammatory response by inhibiting the release of pro-inflammatory cytokines, such as TNF-α and IL-6, and by promoting the production of anti-inflammatory cytokines, such as IL-10, contributing to the maintenance of cutaneous immunohomeostasis. At the extracellular matrix level, melatonin stimulates the synthesis of collagen and elastin, essential structural components of the skin, and enhances the organization of collagen fibers, reducing wrinkle formation and improving skin firmness [21,22].

UV radiation can induce DNA lesions, including cyclobutane pyrimidine dimers (CPDs) and 6-4 photoproducts, which can lead to mutations if not effectively repaired. Melatonin has been shown to enhance DNA repair mechanisms, particularly nucleotide excision repair, the primary pathway for repairing UV-induced DNA damage. By facilitating the removal of DNA lesions, melatonin reduces the accumulation of mutations and decreases the risk of skin cancer development [21].

### 2.2. Melatonin and Wound Healing: Promotion of Tissue Regeneration

Beyond its hormonal functions, melatonin exhibits significant biological activities including potent antioxidant and anti-inflammatory properties, infection control, regulation of vascularization and angiogenesis, as well as analgesic and anti-pruritic effects. Research indicates that melatonin can accelerate wound closure and improve tissue regeneration [25]. To overcome limitations like poor water solubility and instability, nanocarrier systems are being explored to enhance their delivery and efficacy in treating skin injuries. Current reviews [26] aim to consolidate scientific evidence supporting melatonin’s therapeutic potential in promoting effective wound healing and its potential application in regenerative medicine and wound dressings.

### 2.3. Melatonin in Skin Cancer Protection

Melatonin exerts direct oncostatic effects on skin cancer cells, inhibiting their proliferation and inducing apoptosis. Melatonin can suppress the growth of melanoma cells by interfering with cell cycle progression and inducing cell cycle arrest [3]. It also activates apoptotic pathways, leading to the elimination of cancerous cells [27].

The mechanisms underlying melatonin’s anti-proliferative and pro-apoptotic effects are complex and involve the modulation of various signaling pathways. Melatonin can influence the expression of oncogenes and tumor suppressor genes, as well as the activity of kinases and transcription factors involved in cell proliferation and survival. Melatonin enhances antitumor immune responses by modulating the activity of immune cells, including natural killer cells, T lymphocytes, and dendritic cells. It can increase the production of cytokines, such as interferon-gamma (IFN-γ) and interleukin-12 (IL-12), which are essential for antitumor immunity. By boosting immune surveillance, melatonin facilitates the recognition and destruction of skin cancer cells, limiting tumor growth and metastasis. Its immunomodulatory properties are particularly relevant in the context of melanoma, which is known to be immunogenic [3,28].

### 2.4. Other Relevant Dermatological Uses: Vitiligo, Rosacea, etc

Vitiligo is characterized by the loss of melanocytes, the melanin-producing cells. Owing to its potent antioxidant properties, melatonin may confer protection to melanocytes against oxidative stress, a major factor for their destruction. Melatonin can stimulate melanocyte proliferation and promote cutaneous repigmentation. Furthermore, its antioxidant action neutralizes free radicals, which play a substantial role in the pathogenesis of vitiligo, and modulates the immune response, implicated in the pathophysiology of this condition [29].

**Figure 1:**
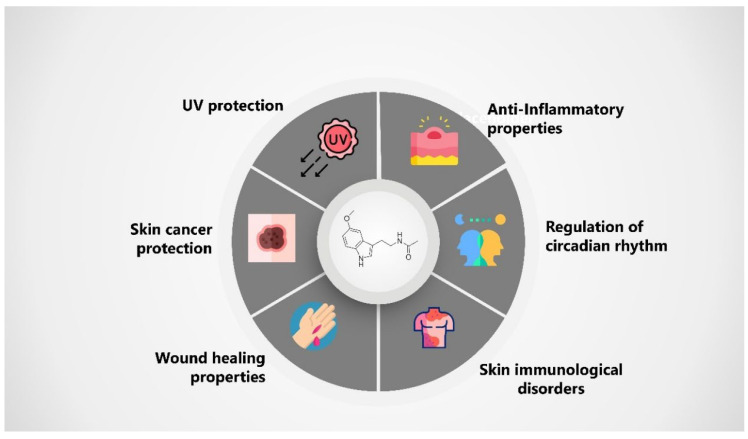
Pharmacological properties and pharmaceutical applications of melatonin.

In rosacea, an inflammatory dermatosis, melatonin possesses anti-inflammatory properties that may mitigate the erythema and inflammation associated with it. It can also protect cutaneous blood vessels, which is beneficial in rosacea [30], where vascular dilation contributes to facial redness. Likewise, melatonin’s antioxidant capability may alleviate oxidative stress, considered a factor in this condition (Figure 1).

## 3. Formulations of Melatonin for Dermatological Use

### 3.1. Topical Formulations: Gels, Creams and Ointments

**Figure 2:**
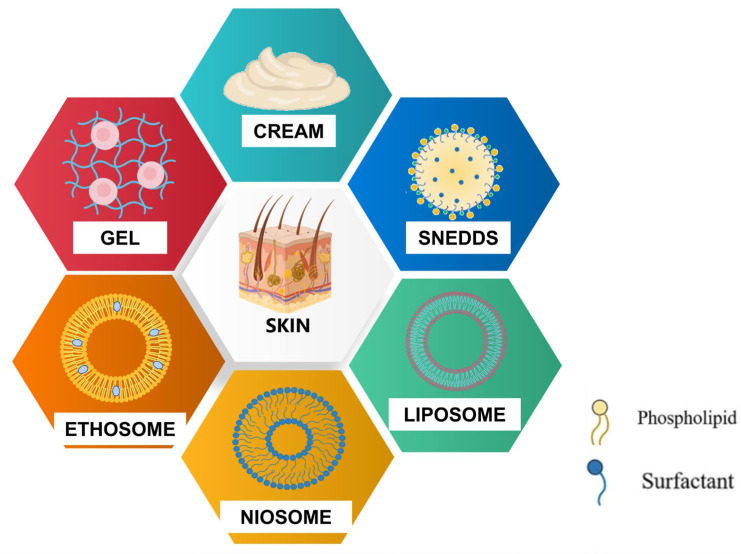
Pharmaceutical technologies utilized for topical delivery of melatonin.

Melatonin, transcending its established role in circadian rhythm regulation, has garnered attention within the dermatological sphere for its inherent antioxidant and anti-inflammatory attributes. This has precipitated the development of topical formulations tailored for a spectrum of cutaneous conditions. The reviewed literature highlights the diversity of melatonin-containing topical and transdermal formulations, spanning from conventional creams to advanced nanosystems. While these studies collectively demonstrate melatonin’s pharmacological versatility, substantial variability exists in experimental design, translational relevance, and mechanistic depth (Table 1 and Figure 2).

#### 3.1.1. Cream Formulations

The compounded melatonin cream using Beeler’s base represents an extemporaneous, clinically tested preparation designed for the prevention and treatment of radiation-induced dermatitis. Clinical outcomes indicated good tolerability and preventive efficacy. Nevertheless, the single-case design, lack of standardized formulation parameters, and absence of quantitative efficacy metrics limit its scientific rigor and industrial reproducibility. The formulation contained melatonin as the sole active ingredient, which facilitates mechanistic interpretation but restricts formulation sophistication.

The night cream containing melatonin, carnosine, niacinamide, hyaluronic acid, matricin peptides, and Helichrysum italicum extract was evaluated in ex vivo and clinical studies, showing significant increases in skin hyaluronic acid content and reduction in photodamage symptoms. However, the presence of multiple bioactive compounds—each with known antioxidant and anti-inflammatory properties—obscures the specific contribution of melatonin to the observed effects. Furthermore, the study design lacked a melatonin-only control, limiting causal inference. Despite positive cosmetic outcomes, the formulation’s complexity and non-pharmaceutical positioning constrain its relevance for therapeutic applications.

#### 3.1.2. Gel-Based Nanoformulations

The niosomal gel developed for the treatment of 5-fluorouracil-induced oral mucositis was investigated through in vivo studies in mice, showing histopathological improvements comparable to corticosteroid formulations. The encapsulation of melatonin within niosomes enhanced mucoadhesion and localized delivery. Nonetheless, the absence of pharmacokinetic profiling and species-dependent differences challenge extrapolation to human mucosal systems.

The ethosomal gel aimed at preventing UV-induced skin damage exhibited over 80% melatonin release in in vitro permeation studies, confirming efficient transdermal delivery. The use of ethanol and lecithin as permeation enhancers significantly improved drug diffusion; however, potential irritation effects and long-term tolerability were not evaluated. The absence of in vivo or clinical validation weakens the translational impact.

The melatonin gel creams designed to study the effect of the oil phase on formulation stability and permeability were examined in vitro. Results indicated improved permeability for oil-free systems, while increased oil content enhanced spreadability and adhesion. These findings underscore the critical balance between diffusion efficiency and formulation rheology. However, the study’s focus on physical parameters rather than biological endpoints limits its pharmaceutical interpretation.

#### 3.1.3. SNEDDS Formulations

Two distinct self-nanoemulsifying drug delivery systems (SNEDDS) were identified. The first, co-loaded with resveratrol and melatonin, was evaluated in vitro for ocular administration. It demonstrated controlled release and improved stability, suggesting potential for treating degenerative ocular diseases. However, the presence of resveratrol, itself a potent antioxidant, introduces ambiguity regarding the primary active contributor to the observed antioxidant and cytoprotective effects.

The second SNEDDS formulation, developed using 3D-printed microfluidic micromixers, demonstrated a 42-fold enhancement in transdermal flux compared to conventional creams, with promising scalability for industrial manufacture. Nonetheless, the study was limited to in vitro testing, and no cytotoxicity or in vivo skin compatibility studies were reported. The formulation’s reliance on surfactants such as Labrasol^®^, Capryol^®^ 90, and Labrafac^®^ may pose irritancy risks that require further validation.

#### 3.1.4. Liposomal and Niosomal Systems

The liposomal melatonin formulation tested in vitro and ex vivo demonstrated moderate improvements (5–10%) in sperm motility, viability, and DNA integrity following cryopreservation. The synergistic effects of melatonin with phospholipids and cholesterol highlight its potential as a cryoprotectant. However, the limited scope and non-clinical application domain (veterinary reproductive biology) restrict the generalizability to broader pharmaceutical contexts.

The niosomal molecular dynamics simulation study provided in silico insights into bilayer organization, showing that melatonin disrupts lipid ordering and promotes lateral expansion within the membrane. While theoretically informative, these results remain computational predictions lacking experimental corroboration. Validation through spectroscopic or calorimetric techniques would be necessary to confirm these molecular mechanisms.

Other topical formulations, including ocular contour products, incorporating melatonin alongside soothing and decongestant agents to diminish fine lines, periocular hyperpigmentation, and edema, thereby enhancing periorbital firmness. Scalp sprays, which stimulate pilosebaceous follicles and promote hair growth, are frequently combined with biotin and adenosine to potentiate their effects. The efficacy of these products is contingent upon melatonin concentration, ingredient synergy, formulation, and vehicle [42].

In summary, melatonin exhibits substantial promise as a multifunctional active agent in topical and transdermal drug delivery systems. Across the reviewed studies, its reported benefits include antioxidant protection, anti-inflammatory activity, enhanced dermal penetration, and cryoprotective effects. However, several critical limitations persist: (i) predominant reliance on in vitro or ex vivo testing with limited in vivo or clinical validation; (ii) complex multi-active formulations that obscure melatonin’s individual role; (iii) lack of standardized methodologies for assessing efficiency and stability; and (iv) insufficient scalability and industrial relevance in most reports.

Future research should prioritize systematic, comparative, and mechanistically driven investigations, incorporating pharmacokinetic analyses, biocompatibility evaluations, and realistic manufacturing approaches such as hot-melt extrusion or microfluidic-assisted assembly. Such advancements are essential to translate melatonin-based formulations from laboratory prototypes to clinically and industrially viable pharmaceutical products.

### 3.2. Melatonin Oral Supplementation for Dermatological Therapy

Oral melatonin formulations are diverse, encompassing tablets, capsules, sublingual tablets, liquids, and gummies, each exhibiting distinct pharmacokinetic profiles [43]. Tablets and capsules, the most prevalent forms, offer immediate or sustained-release options, influencing absorption and duration of effect [44]. Commercially available doses typically range from 1 mg to 10 mg. Sublingual tablets provide rapid absorption, advantageous for dysphagic patients, with doses generally ranging from 1 mg to 5 mg. Liquid formulations enable precise dose titration, crucial for pediatric or individualized dosing, allowing for ranges from 0.5 mg to 10 mg. Gummies enhance palatability, improving compliance, with common doses of 1 mg, 3 mg, and 5 mg. Optimal dosage is patient-specific, necessitating adherence to manufacturer guidelines or healthcare professional recommendations. For insomnia, 1–5 mg is often recommended 30–60 min before bedtime; for jet lag, 0.5–5 mg may be effective, with timing dependent on time zone; and for circadian rhythm disorders, individualized dosing under medical supervision is required. Product quality varies, emphasizing the importance of reputable manufacturers. Melatonin interacts with certain medications, requiring comprehensive medication reconciliation. While generally safe, adverse effects like daytime somnolence and headaches may occur. Clinical applications include insomnia, jet lag, and circadian rhythm disorders [45,46].

Currently, no oral melatonin products are specifically approved for dermatological conditions, and thus, any reported dermatological benefits associated with oral administration are considered off-label uses. However, a growing body of evidence suggests that systemic melatonin supplementation may exert indirect dermatological benefits through its antioxidant, anti-inflammatory, and photoprotective properties. Several studies have explored the off-label use of oral melatonin for conditions such as photoaging, cutaneous melanoma adjuvant therapy, atopic dermatitis, and psoriasis, primarily aiming to modulate systemic oxidative stress and inflammatory pathways rather than to deliver melatonin directly to the skin [2,3,47]. Nonetheless, these investigations are largely exploratory, typically involving small sample sizes and variable dosing regimens, and thus do not constitute formal clinical validation for skin-related indications.

### 3.3. Topical vs. Oral Bioavailability of Melatonin

Melatonin, is primarily administered orally. However, oral bioavailability is relatively low (approximately 15%) due to first-pass hepatic metabolism. Absorption can be influenced by the presence of food, which may delay absorption and decrease peak plasma concentrations. Immediate-release formulations achieve maximum plasma concentrations within 30–60 min, while sustained-release formulations release melatonin gradually over several hours. Sublingual administration allows for rapid and direct absorption into systemic circulation, bypassing first-pass metabolism and potentially yielding higher bioavailability and faster onset of action. Topical melatonin is absorbed through the skin, but systemic absorption is limited, rendering it primarily suitable for local dermatological applications. Factors affecting melatonin absorption include first-pass metabolism, food intake, age-related changes, and interindividual variability. Clinical considerations necessitate careful selection of administration route and formulation based on clinical indication and patient needs. Immediate-release formulations may be more effective for sleep onset, while sustained-release formulations may aid in sleep maintenance. Sublingual melatonin offers an alternative for patients with dysphagia or those seeking rapid action. Topical melatonin is confined to local dermatological applications [44].

The prolonged-release formulation of Circadin is specifically designed to facilitate a more gradual release of melatonin, aiming to mimic the physiological nocturnal secretion profile of endogenous melatonin and sustain plasma concentrations within a pharmacologically relevant range throughout the night [48].

Comparative pharmacokinetic studies have indicated that the C_max_ of melatonin following the ingestion of the prolonged-release tablet is approximately half of that observed with a rapid-release sublingual spray formulation. However, the prolonged-release tablet effectively maintains plasma melatonin concentrations above the physiological threshold (exceeding 100 pg/mL) for up to 6 h post-administration. Endogenous plasma melatonin concentrations exhibit a diurnal rhythm, with low levels during the daytime (around 10 pg/mL) and a nocturnal surge peaking at approximately 40–100 pg/mL between 02:00 and 04:00 h. The administration of Circadin (2 mg) results in supraphysiological plasma melatonin concentrations. One study reported a mean C_max_ of approximately 1200 pg/mL following Circadin administration [48,49]. Bearing in mind the poor oral bioavailability of melatonin, topical formulations can overcome this challenge [50].

Topical Permeability of Melatonin

The topical permeability of melatonin and the systemic plasma concentrations achieved following its cutaneous application are critical parameters for determining its efficacy in dermatological or even systemic applications.

- Lipophilicity: Melatonin exhibits lipophilic properties, which facilitates its permeation through the stratum corneum, the outermost and lipid-rich layer of the skin [51].
- Concentration and Formulation: Permeability is significantly influenced by the melatonin concentration in the topical and by the vehicle (oils), excipients, and penetration enhancers. In particular, self-nanoemulsifying drug delivery systems (SNEDDS)—isotropic mixes of an oil phase, surfactant, and co-surfactant that spontaneously form oil-in-water nanoemulsions on contact with skin moisture—can markedly boost skin transport. Typical SNEDDS generate 20–200 nm droplets, which increases interfacial area, keeps melatonin solubilized (higher thermodynamic activity), and helps surfactant/oil components fluidize stratum-corneum lipids; all of this favors permeation compared with conventional creams [37]. Formulation levers include the oil phase (e.g., medium-chain triglycerides, oleic-acid/Capryol-type lipids), non-ionic surfactants (e.g., Tween 80, Cremophor RH40, Solutol HS15), and co-solvents (e.g., Transcutol P, propylene glycol, PEG-400, ethanol). For dermal targets, SNEDDS are often thickened into gels (SNEDDS-in-gel) to improve residence time and shift the balance toward epidermal/dermal deposition rather than systemic flux; oil-phase choice also materially impacts melatonin stability and permeability [52].
- Skin Condition: The integrity of the skin barrier (e.g., compromised or hydrated skin) can modulate absorption. Notably, skin permeability increases during the nocturnal period, potentially optimizing the application of topical melatonin at night [53].
- In vitro and in vivo Studies: Investigations have shown that melatonin can penetrate human skin and reach deeper layers, with some degree of systemic absorption observed [54]. Across Franz-cell and tape-stripping experiments using human/porcine skin, melatonin crosses the stratum corneum, partitions into deeper layers, and often shows high stratum-corneum retention consistent with controlled release from optimized vehicles. In humans, classic pharmacokinetic studies demonstrated measurable rises in serum melatonin after topical application (cream or alcoholic solution), with vehicle- and dose-dependent profiles; increases were detectable within 1–8 h and generally remained within physiological ranges [55,56]. Transdermal patches in healthy volunteers further confirm systemic absorption, producing a steady plasma rise over ~6–8 h (peak around 8.6 h) together with sleep-maintenance benefits—evidence that dermal delivery can sustain levels over time. Preclinical work also suggests time-of-day effects on transdermal pharmacokinetics, highlighting that absorption and bioavailability can vary with circadian phase [56].

Systemic Plasma Concentrations Achieved [57,58,59]:

- Studies with Creams and Solutions: A study investigating the percutaneous penetration of 0.01% melatonin cream and 0.01% and 0.03% alcoholic solutions in volunteers reported an increase in serum melatonin levels in all cases, although these levels remained within the physiological range.
  ○ The 0.01% cream resulted in a gradual increase, reaching a mean of 9.0 pg/mL at 24 h.
  ○ The 0.01% solution showed an increase to a mean of 12.7 pg/mL at 24 h.
  ○ The 0.03% solution exhibited earlier peaks of 18.1 pg/mL at 1 h and 19.0 pg/mL at 8 h.
- Studies with Higher Doses: Another study employing topical doses of 20 mg and 100 mg melatonin in a 70% ethanolic solution demonstrated significant elevations in serum melatonin levels, with peak concentrations exceeding baseline physiological daytime levels (mean of 16.9 pg/mL in participants). Maximum concentrations displayed considerable inter-individual variability, reaching several thousand pg/mL in some individuals and remaining elevated throughout the 8 h observation period [55].
- Considerations: The extent of systemic melatonin exposure following topical application is generally lower compared to oral administration due to the absence of first-pass hepatic metabolism. However, detectable and potentially systemic effects can occur, contingent upon the applied dose and the formulation characteristics.

In summary, melatonin can permeate the skin and enter the systemic circulation following topical application. The resulting plasma concentrations are dependent on the product’s concentration, formulation, application area, and individual skin characteristics. While concentrations may remain within the physiological range in some instances, higher doses or optimized formulations can lead to supraphysiological levels. This suggests that topical melatonin application exerts not only local effects on the skin (e.g., antioxidant activity) but may also contribute to systemic hormone levels.

Topically applied melatonin generally results in lower systemic exposure compared to oral administration, primarily due to the absence of first-pass hepatic metabolism. Nonetheless, measurable systemic effects may still occur, depending on the applied dose and the physicochemical properties of the formulation. It is important to note that several published studies investigating topical or systemic melatonin have employed doses far above physiological levels, often in the milligram-to-millimolar range. Such concentrations may not reflect achievable or pharmacodynamically relevant exposure in vivo, especially given melatonin’s short half-life and extensive first-pass metabolism. In some reports (e.g., topical application of 20–100 mg melatonin in ethanolic solutions), the observed increases in plasma melatonin are likely a direct result of supraphysiological dosing and enhanced dermal absorption, rather than extraordinary pharmacological potency. Moreover, most available studies rely on placebo controls, whereas comparative evaluation against other indole-based molecules (such as tryptophan, serotonin, or indole-3-acetic acid) could better contextualize melatonin’s unique or shared bioactivities. These limitations highlight the need for standardized dosing regimens, pharmacokinetic analyses, and mechanistic controls to delineate true melatonin-specific effects from nonspecific biochemical responses.

## 4. Clinical and Preclinical Evidence

Preclinical studies, including in vitro and animal models, have provided compelling evidence for the protective effects of melatonin against skin cancer. Melatonin has been shown to inhibit the development of UV-induced skin tumors in mice and to suppress the growth of melanoma xenografts.

While clinical trials are still limited, some studies have suggested that melatonin may have a beneficial effect on patients with skin cancer [60,61]. For example, some studies have investigated the role of melatonin as an adjuvant therapy in melanoma, exploring its potential to enhance the efficacy of conventional treatments. However, more extensive clinical trials are needed to fully evaluate the efficacy and safety of melatonin in skin cancer prevention and treatment. These trials should investigate the optimal dosage, route of administration, and duration of melatonin therapy, as well as its potential interactions with other treatments.

It is a well-established fact that melatonin exhibits remarkably low toxicity. Numerous preclinical toxicology studies have consistently demonstrated that even at significantly elevated doses, an LD_50_ for melatonin could not be determined. This indicates that doses vastly exceeding therapeutic levels did not result in lethality in the animal models investigated [62]. The absence of a defined LD_50_ for melatonin underscores its wide margin of safety. Consequently, the risk of severe, life-threatening overdose intoxication is virtually negligible, a characteristic that differentiates it significantly from many other pharmacological agents. However, it is crucial to note that while not life-threatening, excessively high doses may still induce undesirable side effects. These typically manifest as an exacerbation of melatonin’s sedative properties, including excessive daytime somnolence, dizziness, nausea, headache, or gastrointestinal disturbances [63]. Melatonin’s acute toxicity profile highlights its high safety margin, as evidenced by the inability to establish an LD_50_, although vigilance for dose-related adverse effects remains important [50].

## 5. Synergistic Effects with Other Therapeutic Agents

Melatonin may exhibit synergistic effects when combined with other therapeutic agents, such as chemotherapy, radiotherapy, and immunotherapy (Table 2). For example, melatonin has been shown to enhance the cytotoxicity of chemotherapeutic drugs against melanoma cells and to protect normal tissues from radiation damage.

Combining melatonin with other antioxidants or immunomodulatory agents may also offer synergistic benefits in skin cancer prevention and treatment. Further research is needed to explore the potential of combination therapies involving melatonin.

## 6. Clinical Ongoing Trials Using Melatonin for Dermatological Applications

Melatonin has been evaluated in a variety of clinical and translational settings, ranging from oncological skin injury to inflammatory and photo-induced conditions (Table 3).

### 6.1. Cancer-Related Conditions and Radiotherapy-Induced Damage

The strongest clinical evidence for topical melatonin originates from trials in oncology patients undergoing radiotherapy, where oxidative stress and inflammation contribute to acute skin injury. A randomized, double-blind, placebo-controlled trial by Zetner et al. (2023) demonstrated that a 25 mg/g melatonin cream markedly reduced both the incidence and severity of acute radiation dermatitis in breast-cancer patients [64]. Beyond radioprotection, melatonin has also been explored as an adjuvant in melanoma therapy, based on its pro-apoptotic and immunomodulatory actions [3]. Ongoing trials such as NCT02190838 are assessing combined melatonin + dacarbazine regimens in metastatic melanoma, reflecting growing interest in its potential antitumor role [67].

### 6.2. Photoprotection and Photoaging

Several clinical and ex vivo studies support melatonin’s role in protecting the skin against ultraviolet (UV) radiation and mitigating photoaging. Topical formulations containing melatonin alone or in combination with antioxidants have demonstrated significant increases in dermal hyaluronic-acid levels and reduced erythema following UV exposure [21]. A recent double-blind, placebo-controlled trial reported that oral 5 mg melatonin improved facial melasma, decreasing oxidative-stress markers and pigmentation severity [47]. Also, the nanoencapsulation of melatonin in hyalurosomes have shown promising efficacy in in vivo studies combating UVB-induced skin damage combining the advantages of the hydrating penetration enhancing and antiaging effects of hyaluronic acid along with the powerful antioxidant effects of melatonin [68].

A recent randomized, prospective study evaluated an innovative “In & Out” anti-aging strategy that combined topical and oral melatonin supplementation in 39 healthy women. Participants received either both topical and oral (melatonin (Group A) or topical treatment alone (Group B) for 84 days. Tablets contain melatonin (0.5 mg/tablet), hyaluronic acid (150 mg/tablet), and apigenin (0.9 mg/tablet) while the topical treatment referred to as 0.1% melatonin-based conventional cream. The combined regimen produced significantly greater improvements in skin moisturization (+23.6% vs. +18.3%) and reduction in wrinkle depth (−18.5% vs. −9.4%; *p* < 0.05) compared with topical treatment alone. Lipidomic analysis revealed enhanced levels of triacylglycerols and ceramides, key components for maintaining epidermal barrier integrity and hydration. These findings suggest that systemic supplementation may complement topical melatonin by restoring skin lipid homeostasis and enhancing antioxidant capacity, thereby offering a more comprehensive approach to managing cutaneous aging [69].

Multiple trials are exploring melatonin-based topical products such as sunscreen adjuncts or alternatives. The MELATOX study (NCT02224937) is a randomized, placebo-controlled, double-blind crossover trial evaluating a 12.5% melatonin cream applied to 80% of the body surface. The goal was to assess percutaneous absorption and the cream’s ability to prevent or reduce solar-induced skin damage. Complementing this, another trial (NCT01873430) is investigating melatonin creams ranging from 0.5% to 12.5% applied prior to sun exposure, focusing on sunburn prevention and melatonin’s protective effect against UV-induced inflammation and DNA damage. These studies underscore melatonin’s potential as a novel topical photoprotective agent, offering an alternative or complement to traditional sunscreen ingredients [70].

### 6.3. Inflammatory and Barrier-Related Disorders

Melatonin has been investigated for its ability to modulate immune and oxidative pathways in inflammatory dermatoses. Preclinical and translational data suggest improvements in psoriasis-like and atopic dermatitis models via down-regulation of Th17/IL-17 signaling and restoration of Th17/Treg balance [2]. Also, melatonin can reshape the skin microbiota in mice with atopic dermatitis, especially propionic acid alleviating the symptoms of this disease [71]. Clinically, mucoadhesive melatonin gels have shown promising effects in oral mucositis induced by 5-fluorouracil, yielding comparable histopathological recovery to corticosteroid treatment [35]. Likewise, a melatonin-enriched periodontal gel improved antioxidant enzyme levels and reduced gingival inflammation in periodontitis patients [61].

### 6.4. Wound Healing and Regenerative Applications

In preclinical and early clinical contexts, melatonin has been shown to accelerate cutaneous wound healing through stimulation of fibroblast proliferation, angiogenesis, and collagen synthesis. Hydrogel-based and polymeric formulations have achieved faster closure rates in diabetic and ischemic wounds [7]. These effects are attributed to melatonin’s dual antioxidant and pro-angiogenic actions, making it a promising adjuvant for regenerative dermatology and advanced wound dressings. Melatonin’s regenerative properties are also being applied to wound care and tissue repair. A registered clinical trial (NCT06421454) is currently evaluating a topical melatonin cream for pressure ulcers, a common and challenging condition in elderly or immobilized patients [72]. By modulating local inflammation, promoting fibroblast proliferation, and enhancing antioxidant defense, melatonin may accelerate wound healing in these high-risk populations.

### 6.5. Other Emerging and Exploratory Uses

Additional studies have explored melatonin for hair-growth stimulation, cryoprotection in reproductive tissues, and adjunctive therapy in acne or seborrheic disorders, though these remain preliminary and often confined to in vitro or ex vivo assessments. Additionally, while not involving a topical formulation, an oral melatonin trial in Brazilian women (NCT03831165) investigates its immune-modulatory and anti-estrogenic effects in the context of genital herpes [73]. Although systemic, the outcomes of this study may inform future development of topical or intravaginal melatonin products targeting mucosal infections and inflammation. Such exploratory work reinforces melatonin’s pleiotropic biological activity but requires further controlled clinical evaluation before translation to clinical practice.

Collectively, these clinical trials reflect a growing trend in repositioning melatonin as a multifunctional topical agent, suitable for a wide range of cutaneous and mucosal applications. From photoprotection and wound healing to radiation dermatitis and circadian rhythm restoration, topical melatonin formulations—particularly in the form of gels, creams, and transdermal patches—are gaining traction as safe, non-invasive therapeutic options. Continued clinical validation will be essential to fully establish dosing strategies, formulation parameters, and long-term safety profiles, paving the way for broader regulatory approval and clinical adoption.

## 7. Future Perspectives

### Clinical Implications

Melatonin stands out as a novel therapeutic agent for managing skin conditions driven by oxidative stress, inflammation, or impaired repair. It may serve as an adjuvant therapy, enhancing conventional treatments in diseases such as skin cancer, atopic dermatitis, and photoaging. Its favorable safety profile, absence of dependence or withdrawal effects, and suitability for chronic use enhance its appeal in dermatological practice. Furthermore, its potential role in photoprotection and anti-aging skincare suggests applicability in both clinical and cosmetic settings. Melatonin also offers a dual benefit in itch-related sleep disorders, improving quality of life in chronic dermatoses.

Despite encouraging findings, further research is essential to translate melatonin’s dermatological potential into routine clinical practice [74,75,76,77,78,79,80,81]:

- Large-Scale Clinical Trials: There is a critical need for well-designed, placebo-controlled, multicenter trials to validate the efficacy, optimal dosing, and long-term safety of both oral and topical melatonin across a range of dermatological indications.
- Mechanistic Insights: A deeper understanding of melatonin’s molecular pathways in different skin cell types and inflammatory contexts will help refine its therapeutic applications.
- Optimized Delivery Systems: Development of advanced topical carriers (e.g., liposomes, hydrogels, nanoparticles) is necessary to improve skin penetration, bioavailability, and stability, particularly in cosmetic and wound-healing applications.
- Combinatorial Therapies: Synergistic use with established antioxidants (e.g., vitamins C and E), retinoids, or anti-inflammatory agents could amplify therapeutic outcomes and broaden clinical indications.
- Endogenous Skin Melatonin System: Investigating the skin’s native melatonin synthesis and metabolism may unlock new targets for modulating local melatonin activity in disease contexts.
- Personalized Dermatology: Research into individual variability in melatonin receptor expression or metabolism could pave the way for precision dermatology, allowing tailored treatment approaches based on patient-specific profiles.

## 8. Conclusions

Melatonin represents a promising adjunctive molecule in dermatology owing to its multifunctional biological actions, including modulation of oxidative stress, inflammation, and circadian regulation within the skin. Preclinical and early clinical studies support its potential utility in protecting against UV-induced damage, enhancing wound repair, and mitigating inflammatory skin conditions. Nevertheless, the current level of evidence remains preliminary, and further controlled clinical trials are required to confirm efficacy, optimal dosing, and formulation strategies.

While melatonin is generally regarded as safe and well-tolerated, potential systemic effects, such as mild sedation or alterations in circadian rhythm, should be considered—particularly when used in higher doses or over extended treatment periods. Moreover, the variability among commercially available formulations and the paucity of pharmacokinetic data warrant caution.

In summary, melatonin should not be viewed as a universal or “miracle” compound but as a promising multifunctional adjunct in dermatological therapy whose topical and systemic effects merit rigorous, mechanism-driven clinical validation. Its integration into evidence-based dermatological practice will depend on further elucidation of its safety profile, pharmacodynamics, and comparative effectiveness relative to established treatments.

## Figures and Tables

**Table 1:** Cutting-edge skin formulation approaches to broaden melatonin’s clinical applications. Key: SNEDDS, Self-Nanoemulsifying Drug Delivery Systems.

Topical Formulation	Article Title	Indications	Efficiency and Limitations	Formulation Components	Ref.
Cream	Compounded Melatonin Cream using Beeler’s base for the Prevention and Treatment of Radiation Dermatitis: A Case Report	Radiation-induced dermatitis (Extemporaneous compounded formulation) (Clinical testing)	Prevention of dermatitis caused by radiation; well-tolerated.	Melatonin Beeler’s base	[31]
Cream	Night Cream Containing Melatonin, Carnosine and Helichrysum italicum Extract Helps Reduce Skin Reactivity and Signs of Photodamage: Ex Vivo and Clinical Studies	Signs of Photodamage (Clinical testing)	Hyaluronic acid levels increased by 70.1–83.6% Skin calming efficacy: 86.7–96.7% of patients	Niacinamide, hyaluronic acid, carnosine, matricins peptides, melatonin and Helichrysum italicum	[32]
Niosomal Gel	Topical Melatonin Niosome Gel for the Treatment of 5-FU-Induced Oral Mucositis in Mice	Oral Mucositis (Formulation development and In vivo studies)	No histopathological significant differences between niosomal gel and corticosteroid gel	Mucoadhesive gel with melatonin encapsulated in niosomes	[33,34,35]
Ethosomal gel	Formulation and evaluation of the topical ethosomal gel of melatonin to prevent UV radiation	Prevention of UV radiation damage (Formulation in vitro development)	Melatonin release across the skin > 80%	Melatonin, ethanol, soya lecithin, cholesterol, Carbopol 934	[36]
Gel cream	Unraveling the Impact of the Oil Phase on the Physicochemical Stability and Skin Permeability of Melatonin Gel Formulations	Enhancement of physical stability of melatonin in topical formulations (Formulation in vitro development)	Better skin permeability for formulations without the oil phase but greater skin adhesion and spreadability at higher oily phase content	Melatonin, Pemulen^®^ TR1, isopropyl myristate, olive oil	[37]
SNEDDS	Resveratrol and Melatonin Self-Nanoemulsifying Drug Delivery Systems (SNEDDS) for Ocular Administration	Degenerative ocular diseases (Formulation in vitro development)	35% of melatonin released in Simulated Ocular Environment at 4 h (prolonged release)	Melatonin, Cremophor^®^ EL, Tween^®^ 80, Tween^®^ 20, Solutol^®^HS15	[38]
SNEDDS	Leveraging 3D-printed microfluidic micromixers for the continuous manufacture of melatonin loaded SNEDDS with enhanced antioxidant activity and skin permeability	Antioxidant prevention against chemical warfare agents (Formulation in vitro development)	42-fold higher steady-state transdermal flux compared to conventional creams, formulation scale up using microfluidic micromixers	Labrasol^®^, Capryol^®^ 90, Labrafac^®^ Lipophile WL 1349, Melatonin	[39]
Liposome	Liposome and melatonin improve post-thawed Angora goat sperm parameters	Prevent the deterioration of sperm parameters and provide the cryoprotective effects on sperm DNA (Formulation in vitro development and Ex vivo studies)	5–10% improvements in post-thawed sperm motility, viability, and acrosome integrity with lipids and melatonin combination	Cholesterol, l-α-phosphatidylcholine, stearylamine and melatonin	[40]
Niosomes	Insight into molecular structures and dynamical properties of niosome bilayers containing melatonin molecules: a molecular dynamics simulation approach	Mucoadhesive Properties (Formulation in vitro development)	Melatonin induces a disorderly bilayer structure and greater lateral expansion, opposite to the cholesterol effect	Span, cholesterol and melatonin	[41]

**Table 2:** Topical formulations and drug delivery systems containing melatonin. Key: SOD, superoxide dismutase; PVP, polyvinyl pyrrolidone; HPMC, hydroxymethylcellulose, P407, poloxamer 407.

Formulation	Melatonin Concentration (%)	Use	Effect	Ref.
Cream	0.25	Dermatitis by radiation	Same anti-inflammatory effect than corticosteroid cream	[64]
Cream	0.01%	N/A	Blood levels: 9 pg/mL 24 post-topical administration	[65]
Cream	Beeler’s base	Dermatitis by radiation	Dermatitis by breast cancer radiation resolved in three weeks	[31]
Gel	1% in Carbopol 934	Periodontitis	Increase in SOD levels, control of ROS production and Stage II of periodontitis	[66]
Cream Gel	0.1% oil phase: olive oil and isopropyl miristate and pemulen as gelling agent	N/A	Cream gels with 20% oil phase increased skin adhesion while gels with 0% oil phase had a higher skin penetration	[37]
Ethosome	0.24–0.36% melatonin in ethosomes in carbomer 394 gel	Technological improvement	249–618 nm ethosomes, skin flux of 13.85 μg/cm^2^/h	[36]
Niosome gel	2% melatonin niosomes in chitosan or PVP used in combination HPMC and P407 as gelling agents	Technological improvement	300–500 nm vesicles with higher interaction with the mucins in the oral mucosa	[41]

**Table 3:** Clinical trials for melatonin use in topical applications. Data obtained from clinicaltrials.gov.

Use	Study Type	Melatonin Focus	Key Findings/Status	Clinical Trial Number
Uveal Melanoma	Randomized, Placebo-Controlled	Prevention of metastasis	Ongoing clinical trials assessing metastasis-free survival	NCT06125353 NCT05502900
Melatonin Patches on Sleep in Urological Surgery	Interventional	Topical bioavailability to improve circadian rhythm	Patches with 2.1 mg of melatonin to re-establish the circadian rhythm post-surgery	NCT06910345
Sun protection	Randomized, Placebo Controlled, Double-blind Crossover Study	Solar skin damage	Melatonin cream at 12.5% applied in 80% of the body surface	NCT02224937
	Randomized, doble-blind study	Sunburn	Cream (0.5–12.5%) applied before sun exposure	NCT01873430
Clinical Trial for the Evaluation of Melatonin in the Treatment of Pressure Ulcers	Randomized	Pressure ulcers	Melatonin cream	NCT06421454
Melatonin Effects on Genital Herpes in Brazilian Women	Randomized	Modulatory action in immune and inflammatory responses in genital herpes	Oral dose of melatonin (3–30 mg/daily) due to antiestrogenic effect	NCT03831165
Acute Radiation Dermatitis	Double blind randomized	Radiation dermatitis	1 g of cream melatonin cream with 25 mg/g	NCT03716583
Melanoma	Randomized	Melanoma	Decrease metabolic immunosuppression when combined with dacarbazin in cases of disseminated melanoma	NCT02190838

## Data Availability

No new data were created or analyzed in this study. Data sharing is not applicable to this article.
